# Health Literacy in Schools? A Systematic Review of Health-Related Interventions Aimed at Disadvantaged Adolescents

**DOI:** 10.3390/children8030176

**Published:** 2021-02-25

**Authors:** Craig Smith, Hannah R. Goss, Johann Issartel, Sarahjane Belton

**Affiliations:** School of Health and Human Performance, Dublin City University, D09 NA55 Dublin, Ireland; Hannah.goss@dcu.ie (H.R.G.); Johann.issartel@dcu.ie (J.I.); Sarahjane.Belton@dcu.ie (S.B.)

**Keywords:** health literacy, adolescent, intervention, socioeconomically disadvantaged, school based

## Abstract

Socioeconomically disadvantaged populations are at greater risk of adopting unhealthy behaviours and developing chronic diseases. Adolescence has been identified as a crucial life stage to develop lifelong healthy behaviours, with schools often suggested as the ideal environment to foster healthy habits. Health literacy (HL) provides a possible solution to promote such healthy behaviours. The aim of this study was to review school-based HL-related interventions targeting socioeconomically disadvantaged adolescents and to identify effective intervention strategies for this population. Searches were performed in six databases. Inclusion criteria included age: 12–16; the implementation of a school-based intervention related to HL aimed at socioeconomically disadvantaged populations; an intervention focused on: physical activity (PA), diet, mental health, substance abuse or sleep. Forty-one articles were included, with the majority focusing on PA and diet (n = 13), PA (*n* = 9) or mental health (*n* = 7). Few interventions focused solely on substance abuse (*n* = 2) or sleep (*n* = 1), and none targeted or assessed HL as an outcome measure. There was huge heterogeneity in study design, outcomes measures and effectiveness reported. Effective intervention strategies were identified that can be used to guide future interventions, including practical learning activities, peer support and approaches targeting the school environment, the parents or that link the intervention to the community.

## 1. Introduction

Morbidity and mortality rates from obesity and non-communicable diseases (NCDs), such as type 2 diabetes and cardiovascular disease, are rapidly rising [1,2]. Poor lifestyle behaviours, including low levels of physical activity (PA), high levels of sedentary behaviour, poor dietary habits, substance abuse, poor sleeping habits and mental illness, are said to be large contributors to the NCD burden [3,4,5]. This not only places a heavy burden on society but also on limited health resources [6].

Furthermore, there is extensive literature describing socioeconomic health inequalities in terms of morbidity and mortality. Populations from socioeconomically disadvantaged backgrounds are less likely to avoid unhealthy behaviours [7], and are consequently more likely to develop chronic diseases [8]. There is, therefore, a growing need for the promotion of a healthy lifestyle as a form of primary prevention, particularly in low socioeconomic populations [9].

The World Health Organisation (WHO) defines health literacy (HL) as the cognitive and social skills which determine the motivation and ability of an individual to gain access to, understand and use information in ways which promote and maintain good health [10]. The concept of HL has evolved from what was initially a focus on an individual’s ability to read and understand health information, to focusing on factors that affect an individual’s knowledge, motivation and competencies in relation to health [11,12]. HL has been identified as a personal resource that empowers an individual to make informed health decisions in everyday life and has been acknowledged as a key determinant of health [13,14]. Numerous studies have reported a positive association between high levels of HL, better lifestyle behaviours and better health outcomes in children and adolescents [15,16]. This is highlighted in the Health Behaviour in School-Aged Children survey, which reported HL as one of the major contributing factors leading to health differences [17]. Research indicates that HL is determined by level of education and socioeconomic indicators, with more affluent individuals reporting higher levels of HL [15,18]. Enhancing HL levels in low socioeconomic populations may, therefore, offer a means to reach greater health equity [19]. The WHO has recognised the potential role of improved HL in preventing and reducing NCDs by empowering citizens to manage their own health [13]. Consequently, the WHO has engaged in numerous actions to promote health through an improvement in HL [13,20], and has identified the educational sector as the most important setting for teaching and learning HL in early life [13].

Adolescence is a time period when individuals begin to achieve greater autonomy [21]. Lifestyle behaviours are developed, established, and ultimately track into adulthood [22]. Adolescence is also a period when health behaviours and social determinants, such as the ability to stay in education, can have lasting impacts on health equity across the life course [23]. Thus, this time period is increasingly recognised as challenging, but also as a window of opportunity to improve HL in order to promote positive health behaviours, and consequently reduce the risk of lifestyle-related diseases [24].

A systematic review investigating approaches to behaviour change interventions in young people from disadvantaged backgrounds found that successful interventions incorporated educational components [25], as education has been proven to improve attitudes, develop HL and change health behaviours in youth [26,27]. Furthermore, lifelong behaviours that are developed during adolescence are influenced by educational, biological, cognitive, and socioecological factors [28]. As a result, the school environment has been identified as the ideal setting for interventions to promote health behaviours and reduce NCD risk [29,30]. In addition, it has been suggested that health and education are synergistic [31]; individuals who are well educated are more likely to maintain good health [32], and students with better health are more likely to attain a higher level of education [33]. This relationship between education and health forms the basis of the WHO’s Health-Promoting Schools (HPS) framework, an approach focused on promoting health by addressing the whole school environment [34]. Creating a healthy school environment can, therefore, benefit not only the health and well-being, but also the academic performance of a student [34].

School-based health interventions, however, have reported mixed levels of success [35]. A lack of connection to the fundamental objectives of the schools may be one possible reason for this [36]. To maximise effectiveness, it has been recommended that school-based interventions adopt comprehensive, integrative approaches to health promotion which target both the school environment, and the individual’s attitudes and behaviours [34]. Interventions should also be developed and implemented with a range of actors (students, school staff and community members) [37] and be tailored specifically to the local context [38] to ensure that the needs of the target population are met. This is particularly important when it comes to socioeconomically disadvantaged populations [39]. Socioeconomically disadvantaged populations are not only more likely to have poorer health behaviours, but they are also more likely to have poorer health outcomes following interventions and implementation of government policy changes, when compared to more affluent groups [40,41]. It has even been suggested that poor outcomes following interventions may actually increase health inequalities [42], further emphasising the need to tailor health interventions for disadvantaged populations; an approach which has been proven effective [43,44].

Although previous work has provided recommendations on effective strategies for the implementation of school-based health interventions in the general population, there is a lack of research focusing on interventions specifically targeting adolescents from socioeconomically disadvantaged backgrounds. Of specific interest are interventions aimed at adolescents aged 12–16, as this is typically a time period where adolescents in Ireland and the United Kingdom attend their junior years of post-primary education, creating a practical and pragmatic timepoint to intervene during adolescence [23]. The aim of this paper is to review HL-related school-based interventions in adolescents from socioeconomically disadvantaged backgrounds and to identify effective intervention strategies to improve HL for this population.

## 2. Materials and Methods

This study was registered with PROSPERO: The international prospective register of systematic reviews (REF: CRD42020184410) and adhered to the reporting guidelines of the Preferred Reporting Items for Systematic Reviews and Meta-Analysis Protocols (PRISMA-P) [45].

### 2.1. Study Selection Criteria

Inclusion:

Studies identified through the literature search were included if they:

1. Included typical adolescents with a reported mean age between 12 and 16 years;
2. Self-reported that the participants were from a socioeconomically disadvantaged (or equivalent) background;
3. Included the implementation of an intervention related to health literacy (increases health knowledge, understanding, awareness, motivation, confidence) in at least one of the following areas: physical activity, sedentary behaviour, dietary habits, sleeping habits, mental health or substance abuse;
4. Included school-based interventions, interventions that could be feasibly implemented in a school setting or interventions that could be linked to a school curriculum;
5. Aimed to increase health knowledge/comprehension, understanding, behaviour, value, well-being, motivation, self-efficacy or self-monitoring in relation to any of the following domains: physical activity, sedentary behaviour, dietary habits, sleeping habits, mental health or substance abuse.

Exclusion:

Studies identified in the literature search were excluded if:

6. They included special populations (e.g., children with learning difficulties, pregnant adolescents, exclusively obese individuals, or those with a specific health condition);
7. The intervention did not include an educational element or a component targeting health literacy (increases health knowledge, understanding, awareness, motivation, confidence);
8. They were book chapters, case studies, student dissertations, conference abstracts, review articles, meta-analyses, editorials, protocol papers or systematic reviews;
9. They were not published in English or in a peer-reviewed journal;
10. The full-text article was not available.

For full the PICO statement, see Appendix A.

### 2.2. Information Sources, Search Strategy and Study Selection

Six electronic databases were searched—MEDLINE/PubMed, ERIC, CINAHL, PsychINFO, and EMBASE—to identify relevant evidence. ‘English’ and ‘peer reviewed’ filters were marked on all searches. The search strategy was developed using Boolean operators (AND/OR), incorporating the relevant terms (Appendix B). The search was not limited to any publication time frame. The search was conducted between June and August 2020. All records were exported to Mendeley reference managing software for screening and all duplicates were removed.

Two reviewers (C.S. and H.R.G.) independently assessed the eligibility of the studies. Following title and abstract screening, full-text copies of potentially relevant studies were obtained and screened for full-text inclusion. In the case of disagreement, a third author (S.B. or J.I.) was contacted for discussion until consensus was reached.

### 2.3. Data Collection

Following the screening process, the data were extracted into table format. Study data relating to study information, sample information, purpose of study, intervention description, measurement technique, reported outcome variables, intervention fidelity and intervention quality was extracted. One reviewer (C.S.) entered the data from the included articles into an evidence table, and the second reviewer (H.R.G.) then examined the articles and edited the table entries as needed for accuracy.

### 2.4. Quality Appraisal

Each study was assessed for the risk of bias using a modified version of the Cochrane Collaboration’s tool for assessing risk of bias [46]. The tool was further adapted to remove performance bias as it was deemed inappropriate for the context of this study. Each study was examined for selection bias, attrition bias, detection bias and reporting bias, and ranked as ‘low risk’ or ‘high risk’ for each. It was marked ‘unclear’ if there was insufficient information to make an assessment and marked ‘not applicable’ if the bias could not be determined based on the design of the study in question. A narrative overview of included interventions is also included.

## 3. Results

### 3.1. Study Selection

**Figure 1:**
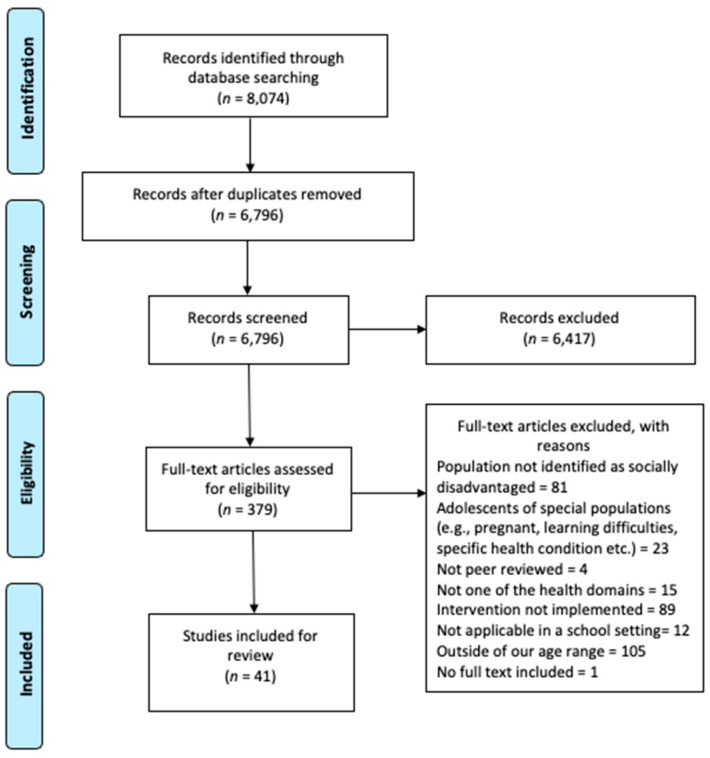
PRISMA Flow Diagram.

Figure 1 below details the search and screening process. The literature search yielded 8074 publications; after removing 1908 duplicates, 6796 publications were subsequently screened. Of these publications, 6417 were excluded based on title and abstract because they did not fulfil one or more of the inclusion criteria. The remaining 379 publications were retrieved for full-text review. A total of 338 failed to meet the inclusion criteria. The main reasons for excluding full texts were that the intervention targeted a population which was outside of the age range, the intervention was not implemented with populations which were socioeconomically disadvantaged. Finally, 41 publications were included for review.

### 3.2. Study Characteristics

The study characteristics are summarised in Table 1 below. Among the 41 studies, 22 were based in the United States [47,48,49,50,51,52,53,54,55,56,57,58,59,60,61,62,63,64,65,66,67,68,69], six in Australia [70,71,72,73,74,75], four in Brazil [76,77,78,79], two in Sweden [80,81], two in India [82,83] and one each in Belgium [84], Chile [85], Spain [86] and Canada [87]. The year of publication ranged from 1996 [59] to 2020 [76], with the majority published in the last decade (*n* = 35). A total of 22 studies were randomised controlled trials (RCTs) [47,48,51,52,55,58,59,63,67,68,70,71,72,73,74,75,76,77,78,82,85,86], ten employed pre-test-post-test quasi-experimental designs [49,53,62,64,65,66,69,79,80,84], four employed a single group pre-test–post-test designs [54,56,61,83], three were post-test qualitative evaluations [57,81,87], and the remaining two adopted cross-sectional designs [50,60]. All studies assessed the interventions using quantitative techniques, with the exception of five which employed mixed methods [49,54,69,86,87], and three that used qualitative analysis [57,60,81]. The sample size ranged from 15 [69] to 13,035 [82]. Twenty-eight of the studies looked at mixed-gender samples, while four focused only on males, and nine targeted females only.

### 3.3. Quality Appraisal

The quality appraisal of the included studies is summarised in Table 2. Many studies were reported as ‘unclear risk’ as they did not provide adequate detail to assess the level of bias. Furthermore, studies which were not RCTs could not be assessed for selection bias as there was no random allocation. Of the studies that were assessed for selection bias, nine reported as ‘low risk’ across both assessments. Due to the lack of information, 15 of studies were reported as having an ‘unclear risk’ of detection bias. Of the remaining studies, seven were reported as ‘high risk’ and 19 as ‘low risk’. The majority (*n* = 35) were scored as ‘low risk’ for attrition bias, with two reported as ‘high risk’. Reporting bias was reported as ‘low risk’ in all included studies.

### 3.4. Intervention Characteristics

The intervention characteristics are detailed in Table 3 below. The largest group of interventions focused on PA and diet (*n* = 13) [49,55,64,65,66,67,69,70,71,77,81,84,86], followed by PA (*n* = 8) [47,48,58,61,73,74,75,76,80] and mental health (*n* = 7) [51,53,56,72,82,83,85]. The remainder targeted diet (*n* = 4) [54,60,62,78]; substance abuse (*n* = 2) [50,68]; PA, diet, substance abuse and mental health [59]; mental health and sleep [64]; PA, diet and substance abuse [52]; Sleep [57]; mental health and PA [87] and PA, diet and mental health [79]. Interventions were delivered by a wide range of people. The duration of the interventions ranged from a single session [55] to a three-year program [72]. Twenty of the interventions incorporated a behavioural theory into the design [47,48,52,54,55,58,61,64,65,66,67,70,71,73,74,75,76,85,87], with 12 of these underpinning their intervention strategies with multiple theories [47,48,54,55,64,65,66,67,73,74,75,76,77,78]. Twenty-three of the 41 interventions did not measure implementation fidelity.

The majority of outcomes associated with the included interventions were concerned with health-related behaviours [50,52,54,55,56,59,61,62,63,64,65,66,68,69,73,74,76,78,80,84,86]. Other outcomes included psychosocial function [51,53,54,55,56,63,68,72,74,78,83,84,85], health-related knowledge [49,52,59,68,69], physical measures (body composition, physical fitness and biochemical measures) [47,59,63,67,70,71,73,77,78] and the cohort’s evaluation of the intervention [57,60,69,81,84,87]. The majority of studies used multi-component interventions (*n* = 30).

### 3.5. Intervention Results

In the interventions targeting both PA and diet, the behavioural outcomes reported were mixed. Five of the interventions improved both PA levels and dietary habits significantly [55,65,66,69], whereas two observed no significant improvement in either behaviour [64,84]. In the interventions aiming to improve PA and nutrition knowledge, both were effective (no significance level reported) [49,69]. Interventions targeting changes in anthropometric measures, such as BMI and body composition, also showed mixed levels of effectiveness. One intervention did not improve any measure of anthropometry [77], while another significantly improved body fat percentage but not BMI [70]. Furthermore, one significantly improved the percentage of individuals who were overweight or obese but did not improve total fat percentage, fat mass or fat free mass of participants [67], and finally Lubans et al. [71] improved BMI and body fat percentage slightly, but not significantly. The two interventions targeting psychosocial correlates associated with PA and dietary habits resulted in significant improvements [55,84]. Interventions which assessed the student’s perception of the intervention displayed positive responses [55,81].

In the interventions targeting PA only, five targeted behavioural outcomes (levels of PA). Four of which were unsuccessful in improving PA levels in their targeted cohorts [58,73,74,80], and one observed a significant improvement in girls but not in boys [61]. Health-related quality of life, however, was significantly improved in one these interventions [74] and muscular fitness in another [73]. Furthermore, interventions focused on reducing screen time did not observe any positive changes in behaviour [76]. Interventions assessing anthropometric measures had higher levels of success, with one study reporting a significant reduction in weight and BMI among the intervention group [75], while another observed a significant reduction in body fat percentage [73] and one observed modest positive changes in body fat percentage [47]. An intervention assessing the dose, reach and fidelity reported positive findings on all three variables [48].

The majority of the mental health interventions targeted psychological functioning, with the authors reporting mixed levels of success. Depressive symptoms were measured in two interventions—one reporting a non-significant improvement [51] and the other finding no intervention effects [85]. In addition, Schleider et al. (2019) [51] reported no improvement in social anxiety symptoms following the intervention. Similarly, Dray et al. [72] found no significant differences between the control and intervention group’s internalising problems, externalising problems and prosocial behaviour scores following the intervention. In a park-based intervention, results showed no significant improvements in parent-reported social skills or problem behaviours. However, staff-reported findings highlighted significant improvements in both problem behaviours and social skills at follow up [56]. In other interventions targeting mental health, social, emotional and academic performance [53], attention and self-efficacy [83], and school climate [82] were significantly increased.

Interventions specifically targeting diet had varied levels of success. Three programs targeted dietary behaviours specifically. One intervention reported a significant increase in the intake of fruit and vegetables and a statistically insignificant decrease in highly processed foods, compared with baseline [54]. Significant increases, however, were seen in psychosocial mediators, and qualitative assessments suggest that the intervention promoted skill building, but environmental barriers made these difficult to use. The two other interventions targeting dietary behaviours reported significant improvements as a result of the interventions [62,78]. School nutrition practice and policy was also significantly improved in the intervention by Alaimo et al. [62]. Finally, one intervention aimed to measure the students’, teachers’ and parents’ perceptions of the intervention, and to identify attributes that were highly valued within [60]. Four key themes ((1) development of life skills, (2) food and health, (3) family and community, and (4) experiential and participatory learning environment) were identified, and all stakeholders positively appraised the intervention.

Both interventions solely targeting substance abuse aimed to improve behavioural outcomes. An intervention by Robinson et al. [50] targeting a reduction in cigarette smoking, alcohol consumption and marijuana use reported significant reductions in cigarette and marijuana use, but not in alcohol consumption. Vicary et al. [68] reported mixed findings on cigarette use and alcohol consumption over the assessment time points in the two intervention groups. Similarly, measurements of skills related to substance abuse (decision making skills, refusal skills, media awareness and resistance skills) resulted in mixed findings.

A multi-health domain intervention which targeted PA, dietary habits, substance abuse and mental health [59] resulted in significantly improved cardiovascular health knowledge scores in males and females. In females, dietary habits, total cholesterol and estimated VO2max also significantly improved. All other risk factors were non-significant in males and females. A sleep intervention qualitatively assessed the acceptability of two mobile phone apps to improve sleep hygiene [57]. The overall feedback on the application was positive, although several barriers were identified, and the students were sceptical about successfully adopting sleep hygiene practices. Furthermore, an intervention targeting both mental health and sleep significantly improved some psychological functioning measures (anxiety, negative coping approaches and self-reported anger) but not cortisol levels or sleep scores [63]. An intervention targeting mental health and PA, which used qualitive analysis to understand the perceived impact of the intervention on youth participants, found that the program was a promising method to improve psychological, social and physical well-being [87]. The adolescents, parents and program implementers described benefits across seven main areas, including dancing and related skills, behaviours (e.g., reduced television viewing), physical well-being, psychological well-being, relationships, respect for others and for diversity, and school performance. A PA, diet and mental health intervention assessed predictors to dropout in the ‘Mexa Se’ intervention [79]. It was reported that in the intervention group, age, body mass, height and BMI were all significant predictors of drop out. Finally, in the intervention implemented by Kerr et al. [52], aimed at improving PA, diet and substance abuse, it was reported that general health knowledge scores increased significantly more in the intervention group compared to the control. Health behaviours, however, did not significantly differ between groups post-intervention.

### 3.6. Effective Intervention Strategies

#### 3.6.1. ‘Hands-On’ or Practical Learning

Of the interventions identified within this review, many adopted ‘hands-on’ or practical learning components, a strategy that proved effective across various health domains. For example, an intervention targeting social, emotional and academic function by Mendelson et al. [53] incorporated mindfulness sessions into the program; resulting in significant improvements across all three health scores (social, emotional and academic function) post-intervention. Similarly, participants in an intervention by Sibinga et al. [63], who practiced mindfulness and yoga within the intervention, reported significantly improved psychological functioning. Furthermore, in an intervention targeting dietary habits [54], participants prepared and ate minimally processed meals with the objective of making these foods look ‘cool and fun’. The intervention resulted in a large significant increase in the participant’s consumption of minimally processed foods. Another example of practical learning was articulated in Jackson et al. [69], where the students learned pertinent nutrition and PA information that was later incorporated into writing and performing their own “healthy” skits in a theatre-based program. This intervention resulted in improved knowledge around PA and dietary habits, improved health-related choices, and the students reported that the intervention was an enjoyable experience.

#### 3.6.2. Peer Support

The use of peer support was another effective intervention strategy identified within this review. For example, a social marketing intervention by Aceves-Martins et al. [63], which aimed to encourage adolescents to increase their fruit and vegetable intake, PA levels and reduce screen time, was led and designed by peer students (who were trained by university specialists). The social marketing intervention resulted in a significant increase in fruit intake, PA levels and a reduction in screen time. Similarly, two interventions [64,66], also targeting dietary habits and PA levels, which used the Transtheoretical Model to tailor intervention strategies and feedback suggestions to adolescents, also adopted a peer-led approach. Participants in the preparation, action and maintenance stages of change were used as peer models for students in the pre-contemplation and contemplation stages of change and led interventions sessions targeting healthy eating and exercise promotion with the assistance of nursing students. Both interventions produced findings supporting the peer-led approach, with one intervention reporting significant changes in dietary behaviour and PA in the intervention group compared to the control [66], and the other reporting non-significant changes in dietary habits and significant changes in PA levels in the intervention group compared to the control [64].

#### 3.6.3. Holistic Approaches

Finally, this review identified the use of holistic approaches to school-based health interventions as an effective strategy. It has been suggested that for health interventions to be effective, they should focus on more than just the educational component [88], with research stating that interventions should also target the school environment and engagement with families or communities (or both) [34]. The benefit of adopting an intervention framework which targets all three areas is demonstrated by Hollis et al. [75]. The intervention, which incorporated teaching strategies to maximise PA and PE lessons (formal health curriculum), the development of school policies and PA lunch programs (school environment), and the development of a community PA expo and community newsletters (community links), reported significant improvements in weight, BMI and PA [89] (these effects were reported in a paper outside of this review). Although no other interventions included in this review met all three of the criteria of the WHO HPS framework as comprehensively as Hollis et al. [75], other interventions which targeted either the community/parent links, or the school environment also reported successful findings. For example, the intervention presented in Casey at al. [74], which was shown to significantly improve health-related quality of life, implemented a PE component that was linked to a community component addressing previously reported barriers to PA participation. Furthermore, the pedagogical approach of the PE program in this study [74] aligned with recent developments in the community sports club. Additionally, a park-based program targeting social emotional learning by engaging the parents through family sessions (supplementing the youth’s sessions) developed specific strategies that the families could model and reinforce at home [56], ultimately leading to significant improvements in the staff reported measures of problem behaviours and social skills in the adolescent’s cohort. Similarly, an intervention by Jackson et al. [69], which was successful in improving PA and dietary knowledge and behaviours (although no significance was reported), involved the parents by hosting healthy eating recipe sessions, completing home-based intervention sessions and inviting the parents to a performance by the adolescents where pertinent dietary or PA information was translated into a theatrical performance. Finally, an intervention by Alaimo et al. [62] involved the assembly of a ‘Coordinated School Health Team’ (CSHT) to improve school nutrition and practices and policies. The CSHT, which was made up of representatives from various sectors of the school (including students), met to discuss school nutrition policies, nutrition environment, health education programs and school food service programs. Intervention schools adopted significantly more nutrition policies and practices than schools in the control group. In addition, students from the intervention schools consumed significantly more fruit and fibre, and less cholesterol than students from the control schools.

## 4. Discussion

The objective of this study was to review the evidence for school-based interventions aimed at HL-related areas in socioeconomically disadvantaged adolescents and to identify effective intervention strategies for this population. To the best of our knowledge, this is the first study to review this topic which is not restricted to a specific region of the world. The evidence collected gives insight into the interventions carried out, and in particular, the success of various strategies implemented with this population, which can be used to guide future intervention development.

Forty-one intervention studies were identified, with the majority carried out in the US. This review aimed to identify studies targeting five health domains (PA, diet, mental health, substance abuse and sleep). The majority of interventions targeted PA, diet and mental health, while very few focused on substance abuse and even less on sleep. This is despite the well-known harmful impacts of substance abuse and poor sleeping habits on adolescent’s health and well-being [90,91,92], and the evidence supporting the benefits of behavioural interventions in improving sleeping habits [93] and substance abuse [92] in youth. Thus, future studies are needed to assess whether interventions targeting sleep and substance abuse are effective in socioeconomically disadvantaged adolescents.

The identified interventions varied greatly in research designs, aims, components, outcome measures and effects, increasing the difficulty to compare and analyse effectiveness per health domain, or across health domains. In addition, although highly recommended [94], only seventeen of the included interventions measured fidelity. This adds to the difficulty of comparing the quality or effectiveness of an intervention, as implementation fidelity of certain intervention strategies within and between studies may have varied greatly. Future studies should include fidelity or process evaluation evidence to allow for a better understanding of the implementation process and to determine whether disappointing results may have been related to poor program delivery [95]. Furthermore, there was a high variance in the risk of bias scores obtained, with numerous interventions scoring as ‘high risk’ or ‘unclear’ on multiple aspects of their study designs, adding to the difficulty of interpreting intervention outcomes. Nevertheless, numerous successful intervention strategies were identified. These include ‘hands-on’ or practical learning, peer support and adopting a holistic intervention approach.

### 4.1. Effective Intervention Strategies

The successful implementation of ‘hands-on’ or practical learning intervention strategies in the studies identified within this review [53,54,63] aligns with previous research that states educational activities which are carried out through interactive tasks and are focused on context specific learning, may improve health-related decision making and motivate adolescents’ to improve behaviour [24,96]. An example of this is ‘LifeLab’ in Southampton; an innovative ‘hands-on’ science-based approach targeting adolescents’ HL through scientific knowledge and lifestyle behaviours [97].

Interventions identified in this review have demonstrated the effectiveness of peer support in adolescent health interventions [63,64,66]. During adolescence, peer relationships begin to develop, and these relationships are reported to have positive or negative influences on health [98]. Connections with supportive and prosocial peers can lead to healthier behaviours and reduced likelihood of risky behaviours [99]. In addition, peer modelling and awareness of peer norms can be protective against behaviours such as sexual risk [100] and substance abuse [101]. The findings from this review have further underlined the benefits of peer support by using adolescent peers to design and deliver intervention components [63,64,66].

This review has provided further evidence for the benefits of adopting a holistic approach to school-based health interventions, rather than just targeting on the curriculum element. The WHO HPS framework suggests that school-based interventions should adopt an eco-holistic approach by targeting three key areas—the formal health curriculum, the school environment, and engagement with families or communities (or both) [34]. While most of the interventions within this review targeted the formal school curriculum dimension of the framework, less were concerned with targeting all three critical elements. The benefit of adopting an intervention framework which targets all three areas is demonstrated by Hollis et al. [75]. Although only one intervention was successful in incorporating all components of the HPS framework [34], others which have targeted critical elements, such as parent or community engagement or the school environment [56,62,69,74], appear to foster positive improvements in the health and well-being of low socioeconomic adolescents.

### 4.2. Implications of the Identified Interventions Strategies for Socioeconomically Disadvantaged Adolescents

As previously mentioned, socioeconomically disadvantaged populations tend to benefit less from health interventions than those from more affluent backgrounds, often resulting in greater health disparities [40]. Yet, to date, there has been limited evidence to inform the design of health interventions for this population [102]. This review, therefore, has attempted to add to the evidence base by identifying effective and attractive intervention strategies which have been shown to work specifically in this socioeconomically disadvantaged adolescent cohort.

Intervention strategies that involve students carrying out practical activities appear to be more effective than those involving didactic learning. This has been reported elsewhere in lifestyle behavioural interventions targeting adolescents from socioeconomically disadvantaged populations, where students have reported enjoying ‘getting away’ rather than learning from the classroom [103]. In addition, providing such ‘hands-on’ activities which are financially inexpensive is crucial when designing intervention strategies for this population, as cost has been reported as being a considerable barrier for participation [70]. The strategies implemented in successful interventions included in this review, such as mindfulness activities [53], yoga [83] and performing “healthy” skits [53], are very cost effective and are therefore suitable options.

Peer learning, in which peers design or deliver (or both) the intervention, appears to be an effective strategy in this cohort. This approach is said to give the students a sense of leadership and empower them to change their lifestyle [104]. Furthermore, including the adolescents in the design and implementation of the intervention is crucial, particularly in a socioeconomically disadvantaged populations, as it ensures the voice of the student is central to the design, which in turn maximises the potential for the intervention to meet the preferences and needs of the population [105]. The WHO strongly advocates the inclusion of peers in the design and implementation of health interventions in socioeconomically disadvantaged populations, as they are trusted by the participants, it leads to greater acceptability and it provides the adolescents with a sense of ownership [106].

Adopting interventions that consist of more than just a school-based educational element appears to be critical in socioeconomically disadvantaged populations. In particular, involving a parents in the intervention as a strategy is important, as parents play a vital role in the lifestyle behaviours of their children [107]. In addition, reaching a range of settings in which youth spend most of their time increases the likelihood of long-term intervention effects [108]. In a review study, the importance of engaging the parents of socioeconomically disadvantaged adolescents in lifestyle behaviour interventions has been highlighted [109], with another study, which failed to change dietary behaviours, stating that the adolescents felt that this was due to a lack of support from their parents [103]. Furthermore, adolescents who perceive their parents to be leading a healthy lifestyle are more likely to also partake in healthy behaviours [110]. Fostering this sort of modelling and parental support through engaging parents in school-based health interventions appears to be a viable solution to improve the impact of interventions in this population.

### 4.3. The Intervention Strategies and Health Literacy

If HL is understood as an observable set of skills, intervention efforts should focus on improving an individual’s skills and capacities [111]. Despite this, previous reviews have indicated that existing HL research has been driven by health concerns, which potentially underplays the development of educational outcomes, such as critical thinking, the development of capabilities and motivation for behaviour change [34,112]. Nevertheless, the current review identifies a number of intervention strategies potentially useful for influencing HL. The classifications of HL provided by Nutbeam (2000) may be a useful way to interpret the strategies identified in the current review [113]. Considering interactive HL, the ability to extract health information, apply new information in changing circumstances and engage with others to make decisions, strategies that include practical learning activities and interaction with peers may be of particular use. Many studies included providing the opportunity for shared decision making as part of the intervention [80,81,82]. Knapp et al. [60] held interactive classes outside of the classroom (in the kitchen and garden). Vicary et al. [68] used a life skills training programme to improve the adolescent’s confidence and capabilities, specifically targeting decision making skills, refusal skills, media awareness and resistance skills in relation to substance abuse. Including the social support network in an intervention may also be of benefit to increase motivation to change behaviour [56,62,74]. Strategies relating to critical HL, the ability to critically analyse information from a wide range of sources, were less prevalent. Only one study [78] attempted to target critical HL by incorporating discussions around a critical approach to the use of supplements in PA. However, many studies used pedagogical techniques and educational resources that could be structured around developing critical HL. For example, discussions [56,59], problem solving [82], role play [52,56], provision of resources [76,78,80], and the development of resilience [72] were all used when targeting health behaviour change. Such components could be easily delivered through practical activities or involve peer interaction to enhance learning. The use of smaller sessions, with groups of similar individuals, to target consciousness raising and self-re-evaluation, with regular feedback could be offered to improve basic functional HL behaviours, which enables individuals to function effectively in everyday situations, as this can improve the motivation and maintenance of behaviour change [66]. Other strategies to consider to improve HL in school aged children may be teacher training [75,76], as existing HL research has suggested pedagogical guidance will be needed to deliver HL informed curricula [14,18,112,114,115], and the holistic approach (previously outlined in this review), which may be a way to incorporate HL as part of the wider HPS framework [14,34,56,62,69,74,75].

It should also be acknowledged that strategies that lead to health behaviour change may or may not lead to improvements in HL and vice versa. One such example of this may be that even in a person considered to have high level of observable HL skills, they may experience real challenges in applying those skills in an unfamiliar environment [111]. This should be considered when interpreting the findings of the current study, which found that none of the included interventions explicitly targeted HL, nor did any use HL as an outcome measure. Despite this, as indicated earlier, strategies may be useful and transfer across HL, health promotion and health behaviours, and future research exploring the transferability of these skills, and the relationship between these connected fields is warranted. A factor to consider in the dearth of school-based HL interventions aimed at socioeconomically disadvantaged populations may be the lack of an appropriate tool to measure HL in this context, with interventions to date using adult-adapted HL tools which some have argued to be inaccurate [116]. As a result, it may have been problematic to place HL as a primary outcome of an intervention. Recently, a tool has been developed specifically for use in adolescent populations, but its applicability is yet to be tested [117]. Furthermore, another HL measurement tool is currently in development, which is following a rigorous co-design process with young people to understand the context and needs of adolescent [118]. The development of valid and sensitive measurement tools can be utilised to guide intervention designs to target the specific needs of a particular population and allow for the potential effects to be easily and accurately tracked.

### 4.4. Applying Effective Intervention Strategies

As adolescents encounter unique health issues based on their level of puberty, development, social environment and social context [22], caution should be exercised when using previous interventions to inform future approaches. ‘Ready-made’ or ‘one-size-fits-all’ health promotion approaches have become increasingly popular [119], yet evidence suggests that interventions developed outside of the targeted schools are rarely meaningful or effective [120]. For health interventions, and in particular HL interventions, to ensure that the health needs and priorities are met, it is recommended that they should be co-produced with all relevant stakeholders [115]. This approach values the knowledge and input of key personnel to facilitate deeper engagement in intervention content, and to ensure that interventions are contextual, sustainable, and equity driven; all of which have been demonstrated in previous HL intervention development approaches [121,122]. Based on this, effective intervention strategies identified within this review can be used to guide the development of future interventions but should always be contextualised and tailored to suit the targeted population.

## 5. Strengths and Limitations

To our knowledge, this is the first study to systematically review school-based HL-related interventions in socioeconomically disadvantaged adolescents. The study provides evidence on interventions carried out globally and was not aimed at a specific region of the world. It must be noted that due to the contentiousness of defining socioeconomically disadvantaged populations, studies which self-identified as socioeconomically disadvantaged (or the equivalent) were included. It is acknowledged that included studies would have used different methods to define socioeconomically disadvantaged populations and, therefore, this may be a limitation when comparing results of studies within this review. The current review used a standardised and well-established appraisal tool for the assessment of study quality to enable comparison between studies. The Cochrane Risk of Bias Tool, however, was originally intended to appraise RCTs [46] and many of the studies included in this review scored poorly or did not report information related to certain aspects of study bias. As a result, the variation between study quality indicates that caution should be exercised when interpreting the results of intervention effects. Due to the large volume of peer-reviewed publications identified and screened in this review, and the practicalities of running and managing this review, the authors did not include a grey literature search. It is acknowledged that by excluding non-published articles, there is an increased risk of publication bias and the possibility of missing out on additional information that a grey literature search may have provided. This review included interventions reporting on outcome and process evaluation, yet many interventions did not report on the process evaluation or intervention fidelity. Future research should consider comprehensively reporting intervention evaluation to provide a deeper insight into the intervention delivery.

## 6. Conclusions

This systematic review provides evidence on the interventions implemented which aimed to improve HL-related areas in socioeconomically disadvantaged adolescents. This review highlights the lack of interventions targeting sleeping habits and substance abuse in this demographic. In addition, no interventions have explicitly aimed to improve HL. Nevertheless, successful interventions strategies were identified that could be used to inform future intervention development. These include the integration of practical-based learning activities and the use of peer educators. Furthermore, evidence supported linking the intervention to the parents and local community.

## Figures and Tables

**Table 1:** Study Characteristics.

Authors (year)	Country	Study Design	Research Type	Sample	Gender
Aceves-Martins et al. (2017) [86]	Spain	RCT	MM	393	M&F
Alaimo et al. (2015) [62]	US	Pre-test-post-test design (quasi-experimental)	QT	1176	M&F
Araya et al. (2013) [85]	Chile	RCT	QT	2512	M&F
Baker et al. (2011) [49]	US	Pre-test-post-test design (quasi-experimental)	MM	46	M&F
Bandeira et al. (2020) [76]	Brazil	RCT	QT	1085	M&F
Beaulac et al. (2011) [87]	Canada	Post-test evaluation	MM	67	M&F
Berria et al. (2018) [79]	Brazil	Pre-test-post-test design (quasi-experimental)	QT	546	M&F
Black et al. (2010) [67]	US	RCT	QT	235	M
Brito Beck da Silva et al. (2015) [78]	Brazil	RCT	QT	833	M&F
Casey et al. (2014) [74]	Australia	RCT	QT	621	F
Dewar et al. (2013) [70]	Australia	RCT	QT	357	F
Dray et al. (2017) [72]	Australia	RCT	QT	3115	M&F
Dubuy et al. (2014) [84]	Belgium	Pre-test-post-test design (quasi-experimental design)	QT	414	M
Fardy et al. (1996) [59]	US	RCT	QT	346	M&F
Frazier et al. (2015) [56]	US	(Single group) Pre-test-post-test design	QT	46	M&F
Frenn et al. (2003) [66]	US	Pre-test-post-test design (quasi-experimental)	QT	117	M&F
Frenn et al. (2003) [64]	US	Pre-test-post-test design (quasi-experimental)	QT	130	M&F
Frenn et al. (2005) [65]	US	Pre-test-post-test design (quasi-experimental)	QT	103	M&F
Fróberg et al. (2018) [80]	Sweden	Pre-test-post-test design (quasi-experimental)	QT	114	M&F
Hollis et al. (2016) [75]	Australia	RCT	QT	1150	M&F
Holmberg et al. (2018) [81]	Sweden	Post-test evaluation	QL	49	M&F
Issner et al. (2017) [55]	US	RCT	QT	100	M&F
Jackson et al. (2010) [69]	US	Pre-test-post-test design (quasi-experimental)	MM	15	M&F
Kerr et al. (2013) [52]	US	RCT	QT	1654	M&F
Knapp et al. (2019) [60]	US	Cross sectional	QL	27	M&F
Leme et al. (2018) [77]	Brazil	RCT	QT	253	F
Lubans et al. (2012) [71]	Australia	RCT	QT	357	F
Luesse et al. (2019) [54]	US	(Single group) Pre-test-post-test design	MM	32	M&F
Mendelson et al. (2015) [53]	US	Pre-test-post-test design (quasi-experimental)	QT	49	M&F
Quante et al. (2019) [57]	US	Post-test qualitative evaluation	QL	27	M&F
Robbins et al. (2016) [48]	US	RCT	QT	1519	F
Robbins et al. (2019) [47]	US	RCT	QT	1519	F
Robinson et al. (2003) [50]	US	Cross sectional	QT	1196	M&F
Romero (2012) [61]	US	(Single group) Pre-test post-test design	QT	73	M&F
Roth et al. (2019) [58]	US	RCT	QT	3763	M&F
Schleider et al. (2019) [51]	US	RCT	QT	222	F
Sethi et al. (2013) [83]	India	(Single group) Pre-test-post-test design	QT	60	F
Shinde et al. (2018) [82]	India	RCT	QT	13,035	M&F
Sibinga et al. (2013) [63]	US	RCT	QT	41	M
Smith et al. (2016) [73]	Australia	RCT	QT	361	M
Vicary et al. (2016) [68]	US	RCT	QT	319	F

(US = United States of America; RCT = randomised control trial; QT = quantitative; QL = qualitative; MM = mixed methods; F = females; M = males; M&F = males and females).

**Table 2:** Quality Appraisal.

Authors	Selection Bias		Detection Bias	Attrition Bias	Reporting Bias
	Random Sequence Generation	Allocation Concealment	Blinding of Outcome data	Incomplete Outcome Reporting	Selective Outcome Reporting
Aceves-Martins et al. [86]	Low risk	High Risk	Unclear Risk	Low risk	Low risk
Alaimo et al. [62]	n/a	n/a	Unclear Risk	High Risk	Low Risk
Araya et al. [85]	Low Risk	Low Risk	Low Risk	Low Risk	Low Risk
Baker et al. [49]	n/a	n/a	Unclear Risk	Low Risk	Low Risk
Bandeira et al. [76]	Unclear Risk	Unclear Risk	High Risk	Low Risk	Low Risk
Beaulac et al. [87]	n/a	n/a	Low Risk	Low Risk	Low Risk
Berria et al. [79]	n/a	n/a	Unclear Risk	Low Risk	Low Risk
Black et al. [67]	Low Risk	Unclear Risk	Low Risk	Low Risk	Low Risk
Brito Beck da Silva et al. [78]	Unclear Risk	Unclear Risk	Unclear Risk	Low Risk	Low Risk
Casey et al. [74]	Low Risk	Unclear Risk	Unclear Risk	High Risk	Low Risk
Dewar et al. [70]	Low Risk	Low Risk	Low Risk	Low Risk	Low Risk
Dray et al. [72]	Low Risk	High Risk	Unclear Risk	Low Risk	Low Risk
Dubuy et al. [84]	n/a	n/a	High Risk	Low Risk	Low Risk
Fardy et al. [59]	Unclear Risk	Unclear Risk	Unclear Risk	Low Risk	Low Risk
Frazier et al. [56]	n/a	n/a	High Risk	Unclear Risk	Low Risk
Frenn et al. [66]	n/a	n/a	Low Risk	Unclear Risk	Low Risk
Frenn et al. [64]	n/a	n/a	Unclear Risk	Low Risk	Low Risk
Frenn et al. [65]	n/a	n/a	Low Risk	Low Risk	Low Risk
Fróberg et al. [80]	n/a	n/a	Unclear Risk	Low Risk	Low Risk
Hollis et al. [75]	Low Risk	Low risk	Low Risk	Low Risk	Low Risk
Holmberg et al. [81]	n/a	n/a	Low Risk	Low Risk	n/a
Issner et al. [55]	Low Risk	Unclear Risk	High Risk	High Risk	Low Risk
Jackson et al. [69]	n/a	n/a	Unclear Risk	Low Risk	Low Risk
Kerr et al. [52]	Low Risk	Low Risk	Low Risk	Low Risk	Low Risk
Knapp et al. [60]	n/a	n/a	High Risk	Low Risk	Low Risk
Leme et al. [77]	Low Risk	Low Risk	Low Risk	Low Risk	Low Risk
Lubans et al. [71]	Low Risk	Low Risk	Low Risk	Low Risk	Low Risk
Luesse et al. [54]	n/a	n/a	High Risk	Low Risk	Low Risk
Mendelson et al. [53]	High Risk	High Risk	High Risk	Unclear Risk	Low Risk
Quante et al. [57]	n/a	n/a	Unclear Risk	Low Risk	Low Risk
Robbins et al. [48]	Low Risk	High Risk	Low Risk	Low Risk	Low Risk
Robbins et al. [47]	Low Risk	High Risk	Low Risk	Low Risk	Low Risk
Robinson et al. [50]	n/a	n/a	Unclear Risk	Low Risk	Low Risk
Romero [61]	n/a	n/a	Low Risk	Low Risk	Low Risk
Roth et al. [58]	Unclear Risk	Unclear Risk	High Risk	Low Risk	Low Risk
Schleider et al. [51]	Low Risk	Low Risk	Unclear Risk	Low Risk	Low Risk
Sethi et al. [83]	n/a	n/a	Low Risk	Low Risk	Low Risk
Shinde et al. [82]	Low Risk	Low Risk	Low Risk	Low Risk	Low Risk
Sibinga et al. [63]	Low Risk	Low Risk	Low Risk	Low Risk	Low Risk
Smith et al. [73]	Low Risk	Low Risk	Low Risk	Low Risk	Low Risk
Vicary et al. [68]	Unclear Risk	Unclear Risk	Unclear Risk	Low Risk	Low Risk

(n/a = not applicable).

**Table 3:** Intervention Characteristics.

	Author	Purpose of the Intervention	Key Features of the Intervention	Delivered by	Duration	Theory	Study Outcome(s) Measured	Effectiveness (in Relation to the Outcome Measures)	Fidelity Measure
**Diet and PA Interventions (*n* = 13)**									
	Aceves-Martins et al. [86]	To increase fruit and vegetable intake and PA, while reducing screen time	(a) Adolescent challenge creator (ACC) training: An initial training session on social media principles and healthy lifestyle theory led by a university specialist in health and communication. (b) Design and implementation of 10 activities: ACCs attended activity design sessions. The themes of the activities were based on the primary and secondary objectives of the study, which would stimulate the interest of their peers and were designed to be attractive. The ACCs presented the intervention in classrooms at the two intervention schools, in which they explained the study, provided social media information, and invited their peers to provide suggestions for activities. The ACCs disseminated the activities using social media platforms, posters and flyers. Information, photographs, and videos pertaining to each activity were uploaded to the campaign’s social media platforms. Number of participants = 170.	ACCs, (trained by university specialists)	12 months	None reported	Fruit and vegetables intake Weekly moderate to vigorous PA (MVPA) Sedentary time (questionnaires)	The percentage of adolescents in the intervention group who consumed ≥1 portion of fruit/day increased by 23.5% (*p* < 0.01). Vegetable consumption differed only in males. The percentage of males consuming ≥1 portion of vegetables/day increased by 27.9% (*p* < 0.01) in the intervention group. The percentage of adolescents in the intervention group who engaged in ≥6 h of PA/week participation increased by 21.2% (*p* < 0.01). The percentage of male adolescents who engaged in ≤2 h of screen time/week increased by 27.9% (*p* < 0.01) in the intervention group and 12.3% in the control group (*p* = 0.01).	No
	Baker et al. [49]	To promote a healthylifestyle and improve healthy behaviours* (oral hygiene, hand washing techniques, PA, personal hygiene, and nutrition and food safety) *information reported in this review relates to PA and nutrition components	Medical students presented a lecture, followed by an exercise session (circuit training), healthy smoothie preparation, and nutritional value of food analysis. The lecture topics were broken down into modules (Physical Health and Healthy Eating Habits), and included methods for achieving optimal fitness, types of exercise, proper weight gaining/loss techniques and nutrition/food safety. The intervention was linked to the NFL Youth Education Town community centre. Number of participants = 46.	Medical students	5 weeks	None reported	Awareness and knowledge aboutexercise and healthy lifestyles Demonstration of effectiveways to exercise and maintain health Awareness andknowledge of proper food handling and better eating habits Demonstration of ways to determine the proper nutritionalvalues of foods (questionnaires)	Physical Health Module: 17% of the participants passed the pre-test (60% or higher). 67% percent of all participants passed their post-test (60% or higher, achieving that objective). 75% of these participants improved their test scores by at least 10%, also achieving that objective. Following the end of the intervention program, the survey showed a positive change in behaviour in 75% of the participants. Healthy Eating Habits Module: 29% of the participants passed the pre-test (60% or higher) .50% of the participants passed the post-test (60% or higher), failing to achieve that objective. 86% of all the participants improved their scores by at least 10%, successfully achieving that objective. The survey following the intervention showed that 50% of the participants positively changed their personal eating habits.	No
	Black et al. [67]	Health promotion and obesity prevention program	A manualised 12-session intervention (“Challenge”) included a rap music video promoting healthy eating and PA principles of mentorship (role modelling and support), participatory learning, goal setting. In addition to setting dietary and PA goals, tracking and evaluating progress, and revising goals as necessary, intervention adolescents prepared and tasted healthy snacks and engaged in PA. Number of participants = 121.	Specially trained, college-enrolled, African American mentors	(Approx.) 10 months	Social Cognitive Theory and Motivational Interviewing	The accretion of body mass (BMI) and body fat % (DEXA)	The percentage of overweight/obese adolescents declined from 54% to 36% in the intervention group, while the percentage declined from 36% to 32% among the control group. The percentage overweight/obese adolescents further declined to 35% in the intervention group, but increased to 38% among the control group at delayed follow up, resulting in a significant difference between groups (χ^2^ = 5.8, *p* = 0.02, GEE). There were no statistically significant interactions between intervention and time in either post-intervention or delayed follow up for total percent body fat, fat mass or fat free mass in the multi-level modelling with the whole sample.	No
	Dewar et al. [70]	To promote PA and healthy eating and prevent obesity among inactive adolescent girls	The Nutrition and Enjoyable Activity for Teen (NEAT) Girls combined a range of strategies to promote lifestyle (e.g., walking to school) and lifetime PA (e.g., resistance training), improve dietary intake, and reduce sedentary behaviours. Intervention components included enhanced school sport sessions and lunchtime PA, nutrition workshops, interactive educational seminars, pedometers for self-monitoring, student handbooks, parent newsletters, text messages to reinforce and encourage health behaviours.Number of participants = 179.	Teachers, researchers, dieticians	12 months	Social Cognitive Theory	BMI	There were no changes in BMI, but there was a group-by-time interaction effect for percentage body fat (–1.96%, *p* = 0.006)	Yes
	Dubuy et al. [84]	To promote a healthy diet and PA	3 components (start clinic, school program, end clinic). Clinics (ran by players): eating healthy breakfast, warm up session with players and signing a lifestyle contract. School element: providing free fruit to all pupils, fruit and vegetable quiz, lessons on importance of drinking enough water, activity breaks and active playgrounds. Number of participants = 268.	Professional soccer players, health workers and teachers	4 months	None reported	PA levels Dietary habits Psychosocial correlates Students’ evaluation of the intervention (questionnaires)	No intervention effects were found for consumption of breakfast, fruit, soft drinks or sweet and savoury snacks. Positive intervention effects were found for self-efficacy for having a daily breakfast (*p* < 0.01), positive attitude towards vegetables consumption (*p* < 0.01) and towards lower soft drink consumption (*p* < 0.001). A trend towards significance (*p* < 0.10) was found for self-efficacy for reaching the PA guidelines. For sports participation, no significant intervention effect was found. 92 students completed the process evaluation questionnaire, the feedback was largely positive.	Yes
	Frenn et al. [66]	To improve the adoption of a diet lower in fat and duration of PA	The primary classroom strategy for the sessions was consciousness raising and self-re-evaluation.Separate smaller group sessions were held for students in the preparation, action, and maintenance stages of change Examples of content used include food pyramids, food diaries, peer leadership, planning exercise sessions. (Specific strategies detailed in the paper.)Number of participants = 60.	Paediatric nursing students	4 classroom intervention sessions (duration of intervention not reported)	Transtheoretical Model and Health Promotion model	Dietary fat intake (questionnaire) PA duration (PA log)	When the Health Promotion/Transtheoretical Model interventions were used in 4 classroom sessions, students had a significantly (*p* <.05) reduced trend toward choosing a diet higher in fat and increased duration of PA, as compared with a control group.	No
	Frenn et al. [64]	To examine improvement related to Healthy People 2010 Objectives (U.S. Department of Health & Human Services, 2000) for low-fat diets and MVPA	Internet and video sessions for students in the precontemplation and contemplation stages of change focused on raising awareness of current eating and exercise, identifying pros (or benefits) of both low-fat diet and exercise, and overcoming cons (or barriers) to consuming low-fat diets and participating in exercise. Those in the precontemplation, action, and maintenance stages of change were prepared as “peer models” and led the healthy snack and exercise labs with the assistance of senior nursing students and faculty. Online feedback was given to all students in the intervention for each internet session. (Specific strategies detailed in the paper.)Number of participants = 67.	Peer models (students), nursing students and other faculty members	Academic year	Transtheoretical Model and Health Promotion model	Dietary fat intake (questionnaire) Weekly MVPA (PA log)	The difference in percentage of dietary fat intake between the intervention and control groups as a whole was not significant. Both control and intervention groups decreased their amount of MVPA, but the level of decrease in MVPA was less among the intervention group (–8.58 min) as compared to the control (–37.61 min; *p* = 0.024)	No
	Frenn et al. [65]	To increase PA and reduce dietary fat among low-income, culturally diverse, 7th-grade students	Eight-session Blackboard platform-delivered internet approach with four 2 to 3 min videos. Sessions included preparing snacks, raising awareness for food early in the day/night-time binging, consciousness raising for PA and caloric balance. Computer-generated tailored feedback based on stage of behaviour change was provided to individual subjects for both PA and dietary fat. (Specific strategies detailed in the paper.)Number of participants = 43.	Blackboard platform delivery (internet and computer based).Previously delivered by paediatric nursing students	1 month	Transtheoretical Model and Health Promotion Model	PA levels (PA log) Dietary fat intake (questionnaire)	Intervention students who completed more than half of sessions increased MVPA by an average of 22 min, compared with a decrease of 46 min for the control group, t 103 = −1.99, *p* = 0.05. Those who completed all three sessions increased PA by 33 min. Those participating more than half the sessions decreased percentage of dietary fat from 30.7 to 29.9, t 87 = 2.73, *p* = 0.008, whereas those in the control had 31.5% dietary fat in pre-test and 31.6% in post-test. Those participating in less than half the diet sessions were not significantly different than students in the control group classes, t 16.6 = −1.843, *p* = 0.08.	None reported
	Holmberg et al. [81]	To improve MVPA, sedentary time, exercise training frequency and duration	The intervention was developed and implemented, as a result of cooperation and shared decision making among the researchers and the participants. Components included health coaching, health promotion sessions and a closed Facebook group. Number of participants = 49.	PhD students and the research team	2 years	None reported	The adolescents’ experiences of participating in a Health-Promoting School-based intervention regarding food and PA, with a focus on empowerment (focus groups)	The adolescents appreciated influencing the components of the intervention and collaborating with peers in active learning activities such as practicing sports and preparing meals. They also reported acquiring new health information, that trying new activities was inspiring, and the use of pedometers and photo-food diaries helped them reflect on their health behaviours. This was echoed by teachers.	No
	Issner et al. [55]	To motivate urban, minority youth to make healthy changes in diet and PA	All participants engaged in goal discussion with a health coach that lasted 3–5 min. The enhanced intervention group continued the intervention after the goalsetting portion wherein facilitators used probes to discuss previous experience, elicit positive change talk, discussion of benefits, and ideas for potential solutions.Number of participants = 51.	“Health coaches” (first and second author and research assistants)	20 min–1 session	Self-Determination Theory and Motivational Interviewing	PA levels Fruit and vegetable intake Autonomous motivation and self-efficacy with an aim to improving healthy behaviours (questionnaires)	There was no significant interaction between the impact of the two intervention conditions on participants’ reports of fruit and vegetable intake across two time periods Wilks Lambda = 1.00, F(2, 52) = 0.19, *p* = 0.66, partial eta squared = 0.004. A main effect of time trended toward significance, Wilks Lambda = 0.95, F(2, 52) = 2.88, *p* = 0.09, partial eta squared = 0.054, with both groups showing an increase in fruit and vegetable intake. There was no significant interaction between participants’ reports of PA across two time periods, Wilks Lambda = 0.99, F(2, 49) = 0.58, *p* = 0.45, partial eta squared = 0.012. Time significantly affected outcomes, Wilks Lambda = 0.90, F(2, 49) = 4.99, *p* = 0.03, partial eta squared = 0.096, with both groups showing an increase in PA at time 2. In the goals only condition, from baseline to follow up, PA significantly increased, t(22) = −2.27, *p* < 0.05 (effect size d = 0.46) and autonomous motivation for PA significantly increased, t(22) = −2.45, *p* < 0.05 (effect size d = 0.56). In the enhanced intervention condition, from baseline to follow up, PA significantly increased, t(22) = −2.17, *p* < 0.05 (effect size d = 0.40), autonomous motivation for diet significantly increased, t(22) = −3.59, *p* < 0.001 (effect size d = 0.67), and self-efficacy for diet significantly increased t(22) = −3.91, *p* < 0.001 (effect size d = 0.67).	Yes
	Jackson et al. [69]	To engage low-income, urban, African American adolescents and their families in learning ways to adopt a healthy lifestyle	Interventions components included nutrition and PA information education, preparation and performance of their own “healthy” skits, team building exercises designed to introduce theatre dynamics, which progressed into script-writing activities, making healthy recipes or snacks. Each session ended with some form of PA (circuit training or a dance routine). At the end of the 6 week program, students performed ‘Getting on Track’ for family and friends. Parents were engaged in three ways: (1) participating in a health information and recipe session, (2) completing home-based activities, and (3) attending the intervention’s culminating event, the Champions of Health Dinner Theatre.Number of participants = 15.	Registered dietitian with a background in theatre and a program assistant	6 weeks	None reported	Food and PA knowledge (questionnaire) Food and PA choices and behaviour (questionnaire) The students experience of the intervention (focus groups)	Increases in the number of participants who knew the daily recommended number of servings of fruits and vegetables as well as the recommended amount of time healthy children should be active. When given a choice between specific food item and activity pairs, participants chose healthier food items and PA over sedentary activity at post-test.An increase in students who responded “sometimes” when asked about healthy behaviour (eating fruits and vegetables instead of sweets and participating in PA instead of watching television) The participants experience of the intervention was positive and identified methods to improve future interventions.	No
	Leme et al. [77]	To help achieve healthy food choices, promote lifestyle and lifetime PA, and reduce screen time activities	Intervention components included enhanced PE classes, PA leadership book, recess PA, weekly nutrition and PA messages delivered by teachers during recess, three interactive seminars led by dietitians, nutrition and PA handbook, nutrition workshops, dietary and PA diaries, parents’ newsletters, text messages to students twice a week to encourage them to be physically active and eat healthily. Number of participants = 142.	Dieticians and teachers	6 months	Social-Cognitive Theory	BMI	No significant effect for BMI (F = 2.120, *p* = 0.135).	Yes
	Lubans et al. [71]	To promote PA and healthy eating and prevent obesity among inactive adolescent girls	The Nutrition and Enjoyable Activity for Teen (NEAT) Girls combined a range of strategies to promote lifestyle (e.g., walking to school) and lifetime PA (e.g., resistance training), improve dietary intake, and reduce sedentary behaviours. Intervention components included enhanced school sport sessions and lunchtime PA, nutrition workshops, interactive educational seminars, pedometers for self-monitoring, student handbooks, parent newsletters, text messages to reinforce and encourage health behaviours. Number of participants = 179.	Teachers, researchers, dieticians	12 months	Social Cognitive Theory	BMI Body fat % (bioelectrical impedance analyser)	After 12 months, changes in BMI (adjusted mean difference, −0.19; 95% CI, −0.70 to 0.33), BMI z score (mean, −0.08; 95% CI, −0.20 to 0.04), and body fat percentage (mean, −1.09; 95% CI, −2.88 to 0.70) were in favour of the intervention, but they were not statistically different from those in the control group.	Yes
**PA (only) Interventions (*n* = 9)**									
	Bandeira et al. [76]	To promote PA and reduce the screen time	Teacher training, which was focused on lifestyle behaviours, including excessive screen time, and its implications for health support material, was delivered to teachers to assist them in organising classes on the topic. Component opportunities were created to encourage PA and decrease screen time in the school environment so that adolescents could play games/sports during free time at school. Supervised sessions of 10–15 min (“Gymnastics at School”) were performed twice a week. Health education messages were spread across the schools, and pamphlets were handed over to students/parents. The messages were also addressed to the psychosocial (self-efficacy, attitude, and social support) and environmental aspects of the practice of PA and reducing sedentary behaviour, especially screen time. Number of participants = 548.	Teachers and school staff, undergraduate PE students	4 months (one semester)	Socio-Ecological Model, Health-Promoting Schools and Social Cognitive Theory	Screen time (questionnaire)	There were no significant differences between intervention and control groups for reduction on screen time, in both sexes (boys: 0.105 h/day, 95% CI: −0.184 to 0.393, *p* = 0.477; girls: −0.065 h/day, 95% CI: −0.383 to 0.252, *p* = 0.686) and age groups (11–13 years: −0.046 h/day, 95% CI: −0.630 to0.538, *p* = 0.878; 14–17 years: 0.193 h/day, 95% CI: −0.077 to 0.464, *p* = 0.162).	Yes
	Casey et al. [74]	To improve health-related quality of life (HRQoL), levels of PA, and a range of potential mediators of PA (e.g., self-efficacy, perceived sport competence).	School PE component which incorporated student-centred teaching approaches and behavioural skill development. The PE component involved students participating in two 6-session units, each designed as one session per week during their ‘normal’ PE class time. The two units were a sport unit (tennis or football) and a recreational unit. The curriculum and teaching approach drew on the principles of Game Sense, an Australian derivative of the Teaching Games for Understanding approach, and productive pedagogies in curriculum development. Number of participants = 362.	PE teachers, community fitness instructors and sports coaches	One academic year	Socio-Ecological Model and Social Cognitive Theory	HRQoL PA levels (questionnaires)	After adjustment for baseline levels of PedsQL, the intervention group had significantly higher scores on all three PedsQL scores: physical functioning (adjusted M ± SE = 83.9 ± 0.7, *p* = 0.005), psychosocial (79.9 ± 0.8, *p* = 0.001) and total score (81.3 ± 0.7, *p* = 0.001)—than the control group (80.9 ± 0.8; 76.1 ± 0.9 and 77.8 ± 0.8, respectively), suggesting that the program positively influenced HRQoL. Differences in PedsQL were also present in the 3-group analysis (intervention completers, intervention non-completers and control), whereby the intervention non-completers had significantly higher scores (84.0 ± 0.8, *p* = 0.021; 80.4 ± 0.9, *p* = 0.003; and 81.7 ± 0.8, *p* = 0.002, respectively) than the control group (80.9 ± 0.8, 76.1 ± 0.9 and 77.8 ± 0.8, respectively). There was no statistically significant difference in either the 2-group or 3-group analysis for mins of leisure time (LT) MVPA, MET-mins of LTMVPA, or in the proportion meeting PA guidelines.	Yes
	Fróberg et al. [80]	To improve MVPA, sedentary time, exercise training frequency and duration	The intervention was developed and implemented as a result of cooperation and shared decision making among the researchers and the participants.Components included health coaching, health promotion sessions and a closed Facebook group. Number of participants = 54.	PhD students	2 years	None reported	MVPA (accelerometer) Sedentary time (accelerometer) Exercise training (ET) frequency and duration (questionnaire)	There were no significant effects on changes in the accelerometer-measured MVPA (β = 0.26, 95% CI = [0.08; 0.43]) and sedentary time (β = −0.19, 95% CI = [−0.55; 0.15]), or the self-reported ET frequency (β = 0.03, 95% CI = [−0.25; 0.33]) and duration (β = 0.27 [95% CI = 0.01;0.60]), among the adolescents	No
	Hollis et al. [75]	To reduce the decline in PA typically observed during adolescence	The intervention components targeted the school curriculum, school environment, and broader community and parental support. School curriculum included teaching strategies to maximise student PA in health and PE lessons, development and monitoring of student PA plans within PE lessons and the implementation of a 10 week enhanced school sports programme.School environment included the development and modification of school policies, PA programmes during school breaks and promotion of community PA providers. Additional interventions strategies included an in-school PA consultant 1 day per week, establishing leadership and support, teacher training resources, teacher prompts and intervention implementation performance feedback to schools. Parent engagement: information was regularly sent to the parents via school newsletters, the school website and newsletters on PA recommendations, school-based PA strategies, promotion of community PA providers and strategies to support their child’s PA. Number of participants = 645.	Teachers	19–24 months (7–9 school terms)	Social-Cognitive Theory and Socio-Ecological Model	Weight (BMI)/BMI z-score Whether any effect was moderated by sex, baseline BMI and baseline PA (accelerometer)	At 12 months, there were group-by-time effects for weight (mean difference (95% CI) =−0.90 kg (−1.50; −0.30), *p* < 0.01) and BMI (−0.28 kg m−2 (−0.50; −0.06), *p* = 0.01) in favour of the intervention group, but not for BMI z-score (−0.05 (−0.11; 0.01), *p* = 0.13). These findings were consistent for weight (−0.62 kg (−1.21; −0.03), *p* = 0.01) and BMI (−0.28 kg m−2 (−0.49; −0.06), *p* = 0.01) at 24 months, with group-by-time effects also found for BMI z-score (−0.08 (−0.14; −0.02), *p* = 0.02) favouring the intervention group. Intervention effects were significant for all adiposity outcomes at 12 and 24 months in both the complete cases and multiple imputation analyses. There was weak evidence of a differential treatment on effect on weight in males compared with females (three-way interaction *p* = 0.22). Among males there, was a statistically significant treatment effect at 24 months in favour of the intervention group (−1.26 kg (−2.11; −0.41), *p* = 0.01). There were no significant effects on weight, BMI and BMI z-score at either 12 or 24 months for females. There was weak evidence of a differential treatment on effect on weight in males compared with females (three-way interaction *p* = 0.22). Among males, there was a statistically significant treatment effect at 24 months in favour of the intervention group (−1.26 kg (−2.11; −0.41), *p* = 0.01). There were no significant effects on weight, BMI and BMI z-score at either 12 or 24 months for females. Weight status at baseline: minimal evidence of differential treatment effects depending on baseline weight for weight (*p* = 0.50), BMI (*p* = 0.57) or BMI z-score (*p* = 0.64). PA level at baseline: no evidence of differential treatment effects depending on activity status at baseline for weight (*p* = 0.94), BMI (*p* = 0.95) or BMI z-score (*p* = 0.31). There was no significant effect on weight, BMI or BMI z-score for either active or inactive students at 12 or 24 months.	Yes
	Robbins et al. [48]	To facilitate long-term attainment of adequate MVPA by enhancing girls’ perceptions of perceived benefits, self-efficacy, enjoyment, social support, role models, autonomy, relatedness, competence and reducing barriers relative to PA	A 90 min PA club included organisational tasks (recording attendance and putting equipment away), healthy snacks, warm up activities, encouragement of MVPA, incorporation of information from the Health Promotion Model and Self-Determination Theory and varying forms of PA. Number of participants = 752	PA club manager and 3–4 PA club instructors	17 weeks	Health Promotion Model and Self-Determination Theory	Dose (accelerometer and observation) Reach (attendance) Fidelity (questionnaire)	Reach: Across the 3 years, the total mean attendance at the PA club was 20.54 ± 16.50 days, equivalent to 41% attendance. 93 evaluations were used to measure dose. Dose Received (exposure): The mean accelerometer measured MVPA time was 21.85 ± 6.16 min, and the average number of steps was 2826 ± 820. Dose Received (satisfaction): 88 of the 93 (95.7%) observations by the process evaluators indicated that the girls liked the PAs conducted in the club, and all agreed that girls liked their club instructors. 451 girls completed the satisfaction questionnaire after the 17-week intervention. On average, 87.8% (*n* = 396) liked the activities offered in the club, and 85.4% (*n* = 385) liked the club coaches/managers. Fidelity: process evaluators perceived that the PA club was well received by the girls and delivered with high quality by the coaches/managers. In addition, girls perceived the club was successful in increasing their PA.	Yes
	Robbins et al. [47]	To facilitate long-term attainment of adequate MVPA by enhancing girls’ perceptions of perceived benefits, self-efficacy, enjoyment, social support, role models, autonomy, relatedness, competence and reducing barriers relative to PA	A 90 min PA club included organisational tasks (recording attendance and putting equipment away), healthy snacks, warm up activities, encouragement of MVPA, incorporation of information from the Health Promotion Model and Self-Determination Theory and varying forms of PA. Number of participants = 752.	PA club manager and 3–4 PA club instructors	17 weeks	Health Promotion Model and Self-Determination Theory	BMI z-scores Body fat % (bioelectrical impedance analyser) Aerobic performance (VO2max)	No significant between-group differences in BMI-z existed at post-intervention, but % body fat increased less among intervention than control group girls (M_change_ = 0.43% vs. 0.73%). Aerobic performance decreased less in intervention vs. control (M_change_ = −0.39 vs. −0.57).	Yes
	Romero [61]	To increase frequency of vigorous PA	The first 20 min of lessons were interactive sessions focused on lesson content followed by 30 min break dancing sessions. Lessons were created in collaboration with key stakeholders (middle school students, middle school teachers, health educators, and local break dancers). Key components of the intervention were based on Social Cognitive Theory and included the following: self-efficacy, culturally similar social role models, positive specific feedback on behaviour by teachers and peers, regular logs of PA, setting measurable goals, and identifying neighbourhood resources for PA. Number of participants = 71.	Bilingual/bicultural female university students	5 weeks	Social Cognitive Theory	Vigorous PA Dance frequency (questionnaire)	For girls, a significant increase in vigorous exercise was found from pre-test to post-test, but this was not significant for boys. No significant differences were found in dance frequency.	No
	Roth et al. [58]	Predisposing, enabling, and reinforcing factors for PA as well as self-reported PA	Intervention schools were provided a middle school PE curriculum, $2500 in equipment vouchers for use in PE classes, and a $200 stipend for completing all 12 h of the training. Number of participants = 3763.	PE teachers	2 years	Social Learning Theory	Daily PA Muscle-strengthening PA (questionnaire)	While there were no detectable intervention effects on daily PA, there was a negative intervention effect detected for weekly muscle strengthening PA.	No
	Smith et al. [73]	To examine the mediating effect of resistance training skill competency on percentage of body fat, muscular fitness and PA	Intervention components included researcher-led seminars for students, provision of fitness equipment to schools, smartphone application and website, pedometers for self-monitoring, parental strategies for reducing screen time (i.e., newsletters), lunch-time PA mentoring sessions and face-to-face activity sessions run by teachers during the timetabled school sport period. Number of participants = 181.	Teachers	20 weeks	Self-Determination Theory and Social Cognitive Theory	Body fat % (bioelectrical impedance analyser) Muscular fitness (hand grip and push-up tests) MVPA (accelerometer)	The mediated effect was statistically significant for percentage of body fat (B [SE] = −0.95 [.26]; 95% CI = −1.49 to −0.47) and muscular fitness (B [SE] = 0.16 [.07]; 95%CI = 0.03 to 0.31). The mediated effect was not significant for MVPA (B [SE] = 0.50 [2.1]; 95%CI = −3.6 to 4.6).	Yes
**Mental Health Interventions (*n* = 7)**									
	Araya et al. [85]	To reduce depressive symptoms among low-income secondary school students	The intervention consisted of 11 weekly and 2 booster sessions, each lasting approximately 1 h. There was an introductory session, 6 sessions dealing with thought restructuring and emotions, 3 sessions of problem-solving strategies, and 1 closing session to revise and integrate all previous work. Two booster sessions delivered at months 2 and 7 reviewed challenging negative thoughts and problem-solving strategies. Number of participants = 1221.	Psychologists, occupational therapists, and social workers)	3 months	Cognitive Behavioural Model	Depressive symptoms (questionnaire)	There was no evidence of any clinically important differences between the intervention and control arms in depressive symptoms scores at 3 months (adjusted difference in means, −0.19; 95% CI, −1.22 to 0.84; *p* = 0.72) or at 12 months. The adjusted difference in the primary outcome at 3 months between trial arms was −0.15 (95% CI, −1.12 to 0.81; *p* = 0.75) with 20 imputed full data sets.	Yes
	Dray et al. [72]	To increase the provision of universal strategies targeting multiple internal and external resilience protective factors	A framework of sixteen intervention strategies. Each strategy was designed to address one or more internal or external resilience protective factor. Intervention schools were asked to meet the prescribed set of strategies; however, schools were given the flexibility to select which specific programs or resources to implement to address each of the strategies. (The 16 strategies are detailed in the paper.)Number of participants = 1909.	Teachers	3 years	None reported	Total Strengths and Difficulties Questionnaire (SDQ) score Internalising problems Externalising problems Prosocial behaviour (questionnaires)	There was no significant difference between intervention and control groups for the outcomes of total SDQ, internalising problems and prosocial behaviour There was a significant difference for the outcome of externalising problems in favour of the control group, though the magnitude of effect was small (b¼0.43, 95% CI: 0.04 to 0.83, *p*¼0.02)	Yes
	Frazier et al. [56]	To leverage recreational activities for social emotional learning	Leaders @ play: The program included didactic instruction, skills demonstration and discussion, role plays, and sports and recreation to provide practice with feedback. The first two sessions included team building activities; introduction to the Good Behaviour Game; and orientation to the junior camp counsellor internship. Intervention content emphasised social problem solving, emotion regulation, and effective communication. The last two sessions included review, celebration, and preparation for summer camp. Families @ Play: Multi-family groups comprised of youth, parents, and extended family were designed to meet twice per month for 90 min. The format and content mirrored those of Leaders @ Play. The primary goal was to introduce a targeted skill (problem solving, emotion regulation, or effective communication), accompanied by specific strategies by which families could model and reinforce them at home. Number of participants = 46.	Physical instructor, park recreation leaders, park supervisors, mental health providers	Leaders @ play = 10 weeks. Parents @ play = 10 weeks	None reported	Social skills Problem behaviour (questionnaire)	There were no significant changes in parent report of Social Skills over time: baseline to post-test: t _62_ = −0.23, *n*.s., post-test to follow up: t _50_ = 1.19, n.s, and baseline to follow up: t _64_ = 1.08, n.s. Despite a trended increase in parent-reported Problem Behaviours from baseline to post-test, t _65_ = −1.84, *p* = 0.56 (Cohen’s d = −0.46), these ratings returned to baseline levels by follow up, t _50_ = 1.83, *p* = 0.07 (post-test to follow up) and t _65_ = 0.26, *n*.s. (baseline to follow up). Staff-reported Problem Behaviours for the total sample across sites showed no change from baseline to post-test, t _49_ = 1.64, n.s., but declined significantly by follow up, t _61_ = 2.04, *p* < 0.05 (post-test to follow up) and t _60_ = 3.75, *p* < 0.0001 (baseline to follow up). Staff-reported Social Skills improved from baseline to post-test, t _51_ = −2.56, *p* = 0.01 and follow up, t _63_ = −2.11, *p* < 0.05, and gains were maintained from post-test to follow up, t _62_ = 1.49, n.s. Effect sizes based on overall means from the total sample showed staff-reported reductions in Problem Behaviours (d = 0.46 at post-test and 0.88 at follow up) and gains in Social Skills (d = −0.72 and −0.53, respectively).	Yes
	Mendelson et al. [53]	To enhance social, emotional, and academic functioning	RAP Club incorporates psychoeducation, cognitive behavioural, and mindfulness strategies from three evidence-supported treatments: Dialectical Behaviour Therapy for Adolescents, Trauma Adaptive Recovery Group Education and Therapy and School-Based Trauma/Grief Group Psychotherapy. Number of participants = 29.	Co-facilitated by a mental health counsellor and young adult community member	6 weeks	None reported	Social, emotional, and academic functioning (questionnaire)	Compared with controls, intervention students improved on teacher rated dysregulation (F(1,43) = 7.94, *p* < 0.01, d = 0.85), social competence (F(1,43) = 8.32, *p* < 0.01, d = 0.87), academic competence (F(1,45) = 6.65, *p* < 0.05, d = 0.76), and authority acceptance (F(1,43) = 5.43, *p* < 0.05, d = 0.69). The pattern of scores was in the predicted direction for all the other teacher-reported outcomes, except attention. Student-reported outcomes did not differ by study condition. 17% of intervention students had elevated baseline depression; all displayed a pattern of reduced post-test symptoms. 83% of intervention participants reported low baseline depression; compared with control participants with low baseline depression, these students showed improved teacher-rated dysregulation (t(39) = 2.9, *p* < 0.01), social competence (t(38) = −2.57, *p* < 0.05), academic competence (t(40) = −2.27, *p* < 0.05), authority acceptance (t(39) = 2.53, *p* < 0.05), and disciplinary sanctions ((t(39) = 2.28, *p* < 0.05) Higher program dose was associated with greater improvement than low dose on teacher-rated academic comparison (t(1,25) = 2.93, *p* < 0.01), discipline (t(1,25) = 2.24, *p* < 0.05), and conduct problems (t(1,25) = 2.4, *p* < 0.05).	No
	Schleider et al. [51]	To reduce depressive symptoms, social anxiety symptoms, and conduct problems	Growing Minds (GM): A self-administered, computerised single-session intervention (SSI) which includes content related to multiple types of mind-sets (personality, intelligence, self-regulation) across four interactive modules. Number of participants = 115.	Self-administered on a computer. Research assistance was available for assistance if needed	1 session	None reported	Depressive symptoms Social anxiety symptoms Conduct problems (questionnaires)	Relative to girls in the control group, girls receiving the GM-SSI reported modest but significantly greater reductions in depressive symptoms (d = 0.23) and likelihood of reporting elevated depressive symptoms (d = 0.29) from baseline to follow up. GM-SSI effects were non-significant for social anxiety symptoms, although a small effect size emerged in the hypothesised direction (d = 0.21), and non-significant for change in conduct problems (d = 0.01).	No
	Sethi et al. [83]	To improve attention and self-efficacy	All the students participated in the yoga course for 5 days. The module was selected from Integrated Approach of Yoga Therapy for positive health. Number of participants = 60.	Not reported	5 days	None reported	Attention (Rosenberg self-esteem scale) Self-efficacy (d2 test)	The intervention resulted in a significant increase in self-efficacy (*p* = 0.001) and attention scores (*p* < 0.001).	No
	Shinde et al. [82]	To improve school climate and health-related outcomes	The intervention identifies four priority areas for action: promoting social skills among adolescents; engaging the school community in school-level decision-making processes; providing access to factual knowledge about health and risk behaviours to the school community; and enhancing problem-solving skills among adolescents. The intervention strategies were organised at three levels—whole school, group, and individual levels—and include school health committee, awareness generation fun activities, speak out box (letterbox), wall magazine, competitions (debates, poster making, quizzes, etc.), health policies, peer groups, workshops, individual counselling. Number of participants in the teacher-led intervention = 4046. Number of participants in the lay counsellor-led intervention = 4524.	Lay counsellor (SM) or teacher (TSM)	One academic year	None reported	School climate (questionnaire)	Participants in the SM-delivered intervention schools had substantially higher school climate scores at endpoint survey than those in the control group (BBSCQ baseline-adjusted mean difference [aMD] 7·57 [95% CI 6·11–9·03]; effect size 1·88 [95% CI 1·44–2·32], *p* < 0·0001) and the TSM-delivered intervention (aMD 7·57 [95% CI 6·06–9·08]; effect size 1·88 [95% CI 1·43–2·34], *p* < 0·0001). There was no effect of the TSM-delivered intervention compared with control (aMD −0·009 [95% CI −1·53 to 1·51], effect size 0·00 [95% CI −0·45 to 0·44], *p* = 0·99).	Yes
**Diet Interventions (n = 4)**									
	Luesse et al. [54]	To increase intake of whole/minimally processed foods, operationalised as fruit and vegetables, and decrease intake of highly processed foods, operationalised as sugar-sweetened beverages, fast foods, and processed-packaged snacks	In ’Defence of Food’ is a health education curriculum of 10 sequential 2 h educational lessons. The lessons were structured into three units, consisting of three lessons each, followed by a final celebration lesson. Key aspects of lessons included food rules, film clips, food preparation/tasting, goal setting.(Detailed curriculum components within the paper.) Number of participants = 32.	After-school program teachers	10 weeks	Social Cognitive Theory and Self-Determination Theory	Intake of whole/minimally processed foods (questionnaire) Intake of highly processed foods (questionnaire) Psychosocial mediators of dietary habits (semi-structured interview) Qualitative evaluation (semi-structured interview)	There was a significant increase in mean frequency of fruit and vegetable intake at post-test compared with pre-test (t = 3.359, *p* < 0.01)—an effect size that is considered to be large (d = 0.59). Small effect sizes (d = 0.34) were seen for mean intakes of highly processed foods but change in score was not statistically significant (*p* = 0.06). Statistically significant increases in outcome expectations and self-efficacy for fruit and vegetable intake occurred from pre-test to post-test; all other mediators showed no statistically significant changes. Youth discussed supports for eating fruit and vegetable intake, including social support and modelling, the application of self-regulation skills to increase intake, and their expressed preferences for fruit and vegetable. Youth were also preoccupied with the negative physical outcome expectations of eating highly processed foods, such as developing diabetes.	No
	Brito Beck da Silva et al. [78]	To promote adequate and healthy eating	Eight meetings lasting 50 min each were provided to promote healthy eating and PA. The topics covered were: 1) healthy eating; 2) PA and sports; 3) fats, sugars and salt: effects of a poor diet; 4) nutritional evaluation: The basics; 5) use of supplements in PA: A critical approach; 6) food labelling: food and nutritional safety; 7) role of nutrients in health promotion: functional foods with a focus on fruits, vegetables and legumes; 8) good food handling practices. A webpage was created in a social network used by the adolescents, in which videos, trivia and general guidelines on healthy eating were posted.For parents and/or guardians, didactic-educational materials were sent through adolescents to encourage them to maintain a healthy lifestyle. Number of participants = 387.	Nutritionists	9 months	None reported	Biochemical profiles (TC, HDLc, TG and LDL) (blood test) Anthropometric data (height and weight) Healthy food consumption (legume and vegetable) (questionnaire)	The intervention group exhibited decreases of 7.64 mg/dL (2.94 mg/dL) in mean TC (*p* = 0.009) and 7.77 mg/dL (2.60 mg/dL) in mean LDLc (*p* = 0.003) and increases of 18% in legume consumption (OR = 1.18; 95% CI 1.03–1.37) and 17% in vegetable consumption (OR = 1.17, 95% CI 1.01–1.35) compared with those who did not undergo intervention at the end of the 9-month follow up. No differences were noted in the anthropometric parameters studied.	No
	Knapp et al. [60]	To address individual, social, and environmental factors that affect dietary behaviours	Interactive, garden and kitchen-based curriculum classes during school hours as well as afterschool programming for students. Students were involved in growing, harvesting, preparing, and eating food. Programming also extends beyond the classroom to involve families, school staff, and community members in activities and events, such as family food nights, open garden days, and parent cooking classes. Number of participants = 27.	Program teachers	Not reported	None reported	Student, parent, and teacher perceptions of the intervention Identification of program attributes that are most highly valued Perceived impact of the program on students (focus groups)	Four primary themes emerged from the focus group data: (1) development of life skills, (2) food and health, (3) family and community, and (4) experiential and participatory learning environment. These core themes and subcategories of the themes were organised into levels of the socioecological model.	No
	Alaimo et al. [62]	To improve school nutrition practices (including nutrition education) and policies, and to improve student dietary intake	Schools were asked to convene a Coordinated School Health Team (CSHT) with representatives from various sectors of the school (administration, faculty, food service, health care, and students).Schools were provided with a trained facilitator to meet with their CSHT on one time to complete the healthy School Action Tool (HSAT) healthy eating and nutrition topic area (questions on the following topics: school nutrition policies, school nutrition environment, school health education programs including nutrition education, and school food service programs). At the end of each module, schools were to identify several “bright ideas” they could implement. Schools were asked to prioritise their goals and received $1000 to implement nutrition education or nutrition marketing activities in their action plans.Number of students unknown (40 schools).	Not reported	2 years	None reported	Adoption of school nutrition practices (including nutrition education) and policies (questionnaire) Student dietary intake (questionnaire)	Schools that completed the HSAT prior to but not during the School Nutrition Advances Kids (SNAK) project reported adopting more nutrition policies than schools that never completed the HSAT or a similar program (2.2 vs. 0.4 nutrition policies).Schools that completed the HSAT at any time (prior to but not during the SNAK, during but not prior to, and both prior and during the SNAK project) reported adopting significantly more nutrition practices than schools that never completed the HSAT or a similar program (6.8, 5.8, 7.0, vs. 1.6 nutrition practices, respectively). Schools that completed a similar assessment or grant program before or after the SNAK project also reported adopting significantly more nutrition practices than schools that never completed the HSAT (4.3 vs. 1.6 nutrition practices). Students in schools that were randomised to complete the HSAT reported consuming significantly more fruit (17.5%) and fibre (4.9%) and less cholesterol (4.2%) than students in the control schools. Students in schools that completed the HSAT during the intervention reported consuming significantly more fruit (20.1%) and fibre (5.1%) and less cholesterol (8.4%) than students in schools that had never completed the HSAT.	No
**Substance Abuse (*n* = 2)**									
	Vicary et al. [68]	To reduce the risk for initiation of substance abuse or reduce increased use in high-risk females	The life skills training (LST) condition, which is usually taught by a limited number of teachers in a series of classes dedicated to substance abuse prevention. The infused (I)-LST condition integrates life skills training and alcohol, tobacco or other drugs (ATOD) information into a variety of the existing grade level subject curricula by the teachers for these subject areas. The goal of such an approach is to make prevention an integral part of the total curriculum. I-LST teachers were trained by university/project staff. Number of participants (LST) = 234. Number of participants (I-LST) = 297.	Teachers	2 years	None reported	Students’ self-report of: Cigarette use Smokeless tobacco use Alcohol consumption Attitude toward ATOD Normative beliefs of peer substance use Knowledge about ATOD Change in program-targeted skills: Decision making skills Communication skills Refusal skills Media awareness Resistance skills Assertiveness Coping with anxiety (questionnaires)	The LST low-risk females reported a significantly lower frequency of alcohol use, less binge-drinking, and less marijuana use, while the I-LST low-risk females reported significantly less cigarette smoking. However, the only significant substance use effect that remained for the low-risk females by the end of 8th grade was [less] cigarette smoking for I-LST.Both LST and I-LST positively affected knowledge of alcohol, tobacco, and other drugs. The I-LST program demonstrated a desirable effect on the normative beliefs of the low-risk females at the end of year two. Among skill variables, treatment effects were found for LST low-risk females for decision making, communication, and coping skills at the end of year one, although, at the end of year two, these effects had disappeared. LST females demonstrated significantly worse media resistance skills at the end of year two. Two skill treatment effects existed for the low-risk I-LST females. At the end of year one, I-LST positively affected decision-making skill; however, this effect was reduced to a non-significant level by the end of year two. The I-LST program resulted in greater coping skills by the end of year two for the low- risk females. More positive results were observed, however, for the females at higher risk, with significant treatment effects found in a number of substance use categories. After the first year of programming, high-risk females in the LST program were less likely to use alcohol (both for any drinking and for binge drinking), marijuana, and inhalants. A significant treatment effect was also shown among high-risk females in the I-LST program for drinking, binge drinking, and marijuana use at the end of the first year. The LST program significantly affected pro drug attitudes, normative beliefs, and knowledge of ATOD myths and realities although the effects did not remain at a significant level by the end of year two.The LST program resulted in two treatment effects for the high-risk females, assertiveness and refusal skills. A significant treatment effect was observed for the high-risk females in the I-LST for normative beliefs by the end of year one, although the effect did not remain through year two. A significant I-LST treatment effect was observed for attitudes toward ATOD and refusal skills at year two among the high-risk females.	No
	Robinson et al. [50]	To reduce substance use within a sample of low-income, inner-city African American adolescents	School-based health centre (SBHC) social workers and health educators conduct schoolwide prevention/education groups during regular scheduled classes, as well as schoolwide special assemblies and health fairs. The SBHCs operate similarly to a typical physician’s office. A student’s initial visit to the SBHC includes a comprehensive physical and mental health assessment. Alcohol and drug prevention and rehabilitation services are provided in the form of classroom-based preventative health education and individual counselling. Number of participants = 598.	A physician specialising inadolescent medicine, a nurse practitioner, a social worker, a medical assistant, and a health educator	6 months for 7th graders and 2 years and 6 months for 9th graders	None reported	Substance use prevalence: cigarettes, alcohol, and marijuana (questionnaire)	For the analysis of cigarette smoking, a significant grade 3 SBHC interaction effect was found, F(1, 585) 5 3.83, *p* 5 0.05. The SBHC students smoked slightly less than non-SBHC students in the 9th grade; but, by 11th grade, SBHC students were smoking significantly less than non-SBHC students.The SBHC 3 grade interaction effect for alcohol use was non-significant, F(1, 586) 5 0.39, *p* 0.50, although students from SBHC schools (M 5 1.32, SD 5 3.24) reported drinking slightly less frequently than students from non-SBHC schools (M 5 1.60, SD 5 3.75), this difference was not significant, F(1586) 5 2.45, *p* 5 0.12 For marijuana, a significant grade 3 SBHC interaction effect was found, F(1, 587) 5 12.72, *p*, 0.001. By 11th grade, marijuana use had significantly decreased among SBHC students while marijuana use among non-SBHC students dramatically increased.	No
**PA, Diet, Substance Abuse and Mental Health Interventions**									
	Fardy et al. [59]	To promote health knowledge and behaviour, coronary risk factors, and cardiovascular (CV) fitness	20–25 min circuit training classes followed by 5 min of health behaviours lecture/discussion (topics included exercise, nutrition, smoking cessation, stress management, heart disease, cancer, and motivation). Student workbook from Stanford Adolescent Heart Health Program and the PA and Teenage Health pilot study. Number of participants = 181.	PE teacher and assisted by undergraduate and graduate PE majors	11 weeks	None reported	Health knowledge (questionnaire) Health behaviour (questionnaire) Coronary risk factors (questionnaire) CV fitness (VO2max)	Cardiovascular health knowledge scores significancy increased in the intervention group, whereas they decreased in the control group. Significant changes in self-reported dietary behaviour were observed in female subjects. Significant changes in risk factors were restricted to lowered total cholesterol in girls. Mean cholesterol values in female subjects decreased from 165 to 149 mg/dl in the treatment group, whereas female controls decreased from 154 to 150 mg/dl. There were no significant differences in blood pressure, obesity, and self-reported PA. Estimated mean CV improved in females from 33 to 38 mL/kg per min (*p* < 0.0001), at heart rates of 176 and 152, respectively, whereas control subjects increased only from 33 to 34 mL/kg per min at heart rates of 178 and 172, respectively. In male subjects, treatment and control groups improved from 43 to 52 mL/kg per min and 41 to 49 mL/kg per min, respectively, although the differences between groups were not significant.	No
**Mental Health and Sleep Interventions**									
	Sibinga et al. [63]	To improve mental health and reduce stress	Mindfulness-based stress reduction (MBSR) programs consist of three components: didactic material related to mindfulness, meditation, yoga, and the mind–body connection; experiential practice of various mindfulness meditations, mindful yoga, and the “body scan” during group meetings and encouragement of home practice; group discussion focused on the application of mindfulness to everyday situations and problem solving related to barriers to effective practice. Number of participants = 22.	A mindfulness instructor	12 weeks	None reported	Psychological functioning (questionnaire) Sleep (sleep diaries and actiwatch) Stress (salivary cortisol)	MBSR participants had less anxiety and a reduction in negative coping approaches (*p* = 0.06). MBSR participants showed an increase in self-reported anger (*p* =0.06). Otherwise, there were no significant differences between groups. Among all participants, there was an association between the mindfulness subscale “act with awareness” and lower anxiety (*p* < 0.01). Among MBSR participants, mindfulness subscales were associated with less self-reported angry temperament (pb0.02) and less anger reactivity (*p* = 0.05). Total cortisol output was not statistically significantly different between groups at baseline or follow up. Overall, cortisol output was higher post-program (*p* = 0.05). There was a trend towards increasing cortisol over time among ‘healthy-topic’ participants (113.6 to 167.5, *p* = 0.07); but not among MBSR participants (128.3 to 138.5, *p* = 0.33).Regression analyses of actigraphy data showed no differences between groups in sleep latency (*p* = 0.29), WASO (*p* = 0.42), or sleep efficiency (*p* = 0.97); also, sleep diaries showed no differences in sleep quality (0.67).	No
**PA, Diet and Substance Abuse**									
	Kerr et al. [52]	To improve dietary behaviours, PA, and substance use knowledge and behaviours	Promoting Health Among Teens (PHAT) is a culturally tailored intervention for African American adolescents, focusing on 3 dimensions of health behaviour (diet, PA and substance abuse) for premature cancer and cardiovascular disease prevention.The intervention interactive learning activities to increase health knowledge, develop health behaviour skills, change attitudes, increase self-efficacy, and explore beliefs regarding personal health behaviours. PHAT utilised cultural pride, goal setting, and instruction in dietary behaviours, PA, nutrition cognition. PHAT was conducted using group facilitation, role playing, games, and classroom multimedia messages. (Specific strategies detailed in the paper.) Number of participants = 834.	Trained interventionists	2 weeks	Social Cognitive Theory	General health knowledge Dietary habits PA levels Substance use behaviours (questionnaires)	PHAT participants had significantly higher knowledge scores than FOY (control) participants (*p* ≤ 0.0001), and the rate of increase in condition was significantly higher among PHAT participants than FOY participants (*p* ≤ 0.0001). Participants with greater general health knowledge for the centred health knowledge variable had significantly higher intercepts for past week fruit consumption (*p* ≤ 0.01), past week vegetable consumption (*p* ≤ 0.0001), past month vegetable consumption (*p* ≤ 0.0001) past week moderate PA (*p* ≤ 0.0001), past week PA to strengthen or tone muscles (*p* ≤ 0.01) lifetime alcohol use (*p* ≤ 0.01), and lifetime marijuana use (*p* ≤ 0.05). Participants with lower general health knowledge had higher intercepts for past month alcohol use (*p* ≤ 0.01) and past month marijuana use (*p* ≤ 0.0001).Participants with lower general health knowledge scores had significantly greater slopes for lifetime alcohol use (*p* ≤ 0.01). The growth curves for moderate PA and PA to strengthen and tone muscles were not significantly different between PHAT and FOY participants. The level of engagement of vigorous PA was not significantly different between experimental conditions; however, the rates of increase for participants in PHAT were higher than those in FOY. There were no significant differences in growth curve results between experimental conditions for all past month substance abuse behaviour variables, lifetime alcohol use, and lifetime tobacco use. There were significantly higher rates of increase for PHAT participants in lifetime marijuana use (*p* < 0.0001). Growth curve modelling indicated that participants in PHAT had significantly more gains in health knowledge than participants in FOY, but the effects on behaviour were modest.	Yes
**Sleep Interventions**									
	Quante et al. [57]	To assess the acceptability of sleep apps	Sleep app use to monitor sleep hygiene. Participants could choose between two commercially available sleep apps: ‘SleepBot’© and ‘SleepTime’©. Number of participants = 12.	App based	2 weeks	None reported	Use of the app Effects of the app on sleep behaviour and quality Challenges with the app Desirable components of the app (interview)	There were several barriers identified in relation to the adoption of sleep hygiene interventions, namely reluctance to follow scheduled sleep routines on weekends and concern about “parting” with electronics at bedtime. Participants were intrigued by the idea of adopting an app-based sleep intervention but were sceptical that they could successfully adopt sleep hygiene practices and were more interested in making changes on school days than on weekends. The overall feedback on two commercial sleep apps was positive, with a good adherence and engagement rate, and perceived health benefits.	No
**Mental Health and PA**									
	Beaulac et al. [87]	To promote psychological, social, and physical well-being	The intervention was developed from a thorough literature review, consultation with youth and parents, and ongoing dialogue with community partners. Emphasis was placed on improving dance skills and on fostering positive relationships with peers and adult role model. Number of participants = 67.	Dance instructors (Culture Shock Canada)	13 weeks	Socio-Ecological Model	The perceived impact of the intervention, in terms of psychological, social, and physical well-being (focus group, interview and questionnaire)	The findings suggested that the community-based intervention was a promising program for the promotion of youth psychological, social, and physical well-being. The adolescents, parents, and/or personnel described benefits across seven main areas, including dancing and related skills, behaviours (e.g., reduced television viewing), physical well-being, psychological well-being, relationships, respect for others and for diversity, and school performance.	Yes
**PA, Diet and Mental Health**									
	Berria et al. [79]	To improve components of fitness and body image	MVPA, strength and flexibility exercises were increased in PE classes Students were encouraged to use recess time actively with the availability of balls and ropes. Educational sessions were provided on PA, health, nutrition and body image Educational resources used included movie, lectures, confection of posters and music, and cooking workshops Parents were invited to a night-time healthy eating meeting. Number of participants = 328.	PE teachers and a nutritionist	13 weeks	None reported	Predictors of intervention dropout: gender, age, socioeconomic status, PA, screen time, dietary habits, health perception, attitudes and self-efficacy toward PA, perception of the school environment, body image and self-esteem (questionnaire)	In the crude analysis for the entire sample and among students with adequate BMI at baseline, there was a greater probability of dropping out with increasing age and BMI. Students classified as overweight were more likely to drop out with increasing age. In the adjusted analysis, the association with age remained for the entire sample, including students with adequate BMI and with overweight. In addition, for the overweight students, participation in the intervention during the afternoon period and the higher socioeconomic status were associated with dropping out of the intervention.	No

## Data Availability

Not applicable.

## Appendix A. PICO Table

**Table:**

	Inclusion Criteria	Exclusion Criteria
Population	Adolescents with a reported mean age between 12 and 16 years from a socioeconomically disadvantaged (or equivalent) background.	Mean age not between 12 and 16 or special populations (e.g., children with learning difficulties, pregnant adolescents, exclusively obese individuals, or those with a specific health condition).
Intervention	The implementation of an intervention related to health literacy (increases health knowledge, understanding, awareness, motivation, confidence) in at least one of the following areas physical activity, sedentary behaviour, dietary habits, sleeping habits, mental health or substance abuse.	The intervention did not include an educational element or a component targeting health literacy (increases health knowledge, understanding, awareness, motivation, confidence).
Context	School-based interventions, interventions that could be feasibly implemented in a school setting, or interventions that could be linked to a school curriculum.	Book chapters, case studies, student dissertations, conference abstracts, review articles, meta-analyses, editorials, protocol papers or systematic reviews.
Outcome	Aimed to increase health knowledge/comprehension, understanding, behaviour, value, well-being, motivation, self-efficacy or self-monitoring in relation any of the following domains: physical activity, sedentary behaviour, dietary habits, sleeping habits, mental health or substance abuse.	Outside of the targeted health domains.
Study Design	Published in English and in a peer-reviewed journal.	Systematic review, meta-analysis or full text not available.

## Appendix B. Search Strategy (For PubMed)

((((health[Title/Abstract] OR “health literacy”[Title/Abstract] OR “health knowledge”[Title/Abstract] OR “health competenc*”[Title/Abstract] OR “health understanding”[Title/Abstract]) AND (“physical activity”[Title/Abstract] OR exercis*[Title/Abstract] OR sedentary[Title/Abstract] OR inactiv*[Title/Abstract] OR “substance abuse”[Title/Abstract] OR smok*[Title/Abstract] OR vaping[Title/Abstract] OR vape*[Title/Abstract] OR “e-cigarette*”[Title/Abstract] OR cigarette*[Title/Abstract] OR tobacco[Title/Abstract] OR drug*[Title/Abstract] OR alcohol*[Title/Abstract] OR “mental health”[Title/Abstract] OR “emotional health”[Title/Abstract] OR “psychological health”[Title/Abstract] OR wellbeing[Title/Abstract] OR “well-being”[Title/Abstract] OR diet*[Title/Abstract] OR “health* food*”[Title/Abstract] OR food*[Title/Abstract] OR nutrition*[Title/Abstract] OR “eating behav*”[Title/Abstract] OR sleep*[Title/Abstract])) AND (intervention*[Title/Abstract] OR change[Title/Abstract] OR program*[Title/Abstract] OR behav*[Title/Abstract] OR lifestyle[Title/Abstract] OR education*[Title/Abstract] OR “health promotion*”[Title/Abstract] OR effect*[Title/Abstract])) AND (youth[Title/Abstract] OR adolescen*[Title/Abstract] OR teen*[Title/Abstract] OR pupil*[Title/Abstract] OR student*[Title/Abstract] OR “young people*”[Title/Abstract])) AND (poverty[Title/Abstract] OR “low income”[Title/Abstract] OR “low-income”[Title/Abstract] OR “economic factor*”[Title/Abstract] OR “social inequali*”[Title/Abstract] OR socioeconomic*[Title/Abstract] OR “social economic*”[Title/Abstract] OR “social-economic*”[Title/Abstract] OR “social class”[Title/Abstract] OR “welfare assist*”[Title/Abstract] OR “social assist*”[Title/Abstract] OR subsidi*[Title/Abstract] OR “economic burden”[Title/Abstract] OR unemploy*[Title/Abstract] OR disenfranchi*[Title/Abstract] OR impoverished[Title/Abstract] OR penniless[Title/Abstract] OR “financially disadvantaged”[Title/Abstract] OR “financially distressed”[Title/Abstract] OR “economically disadvantaged”[Title/Abstract] OR “socially disadvantaged”[Title/Abstract] OR “health literacy”[Title/Abstract] OR “health proficien*”[Title/Abstract] OR “health inequalit*”[Title/Abstract] OR “health equit*”[Title/Abstract] OR “health qualit*”[Title/Abstract] OR “health inequit*”[Title/Abstract] OR “health disparit*”[Title/Abstract])
